# Inflammation, immune suppression, and tumor progression

**DOI:** 10.1186/1753-6561-7-S2-K20

**Published:** 2013-04-04

**Authors:** Suzanne Ostrand-Rosenberg

**Affiliations:** 1 University of Maryland Baltimore County, Dept. Biological Sciences, Baltimore, MD 21250, USA

## 

The tumor microenvironment is a complex milieu of tumor and host cells. Host cells can include tumor-reactive T cells capable of killing tumor cells. However, more frequently tumor and host components interact to generate a highly immune suppressive environment that frustrates T cell cytotoxicity and promotes tumor progression through a variety of immune and non-immune mechanisms. Myeloid-derived suppressor cells (MDSC) are a major host component contributing to the immune suppressive environment. MDSC accumulate in most patients and experimental mice with cancer. They inhibit both adaptive and innate immunity through a diverse array of suppressive mechanisms and therefore are a significant obstacle for natural immunity and for active cancer immunotherapies. Their accumulation and suppressive potency are driven by pro-inflammatory mediators. In addition to their inherent immune suppressive function, MDSC amplify the immune suppressive activity of macrophages and dendritic cells via cross-talk which results in the up-regulation of inflammatory mediators. Cross-talk between MDSC and other myeloid cells is itself enhanced by inflammation, resulting in an autocrine tumor microenvironment that sustains and amplifies immune suppression. This talk will describe the cell-cell interactions used by MDSC to inhibit anti-tumor immunity and promote tumor progression, and the role of inflammation in promoting cross-talk between MDSC and other cells in the tumor microenvironment.

## Competing interests

There are no competing interests in this presentation.

